# Association between sarcopenia and oral functions in community‐dwelling older adults: A cross‐sectional study

**DOI:** 10.1002/jcsm.13145

**Published:** 2022-12-05

**Authors:** Yoshihiro Kugimiya, Masanori Iwasaki, Yuki Ohara, Keiko Motokawa, Ayako Edahiro, Maki Shirobe, Yutaka Watanabe, Yu Taniguchi, Satoshi Seino, Takumi Abe, Shuichi Obuchi, Hisashi Kawai, Takeshi Kera, Yoshinori Fujiwara, Akihiko Kitamura, Kazushige Ihara, Hunkyung Kim, Shoji Shinkai, Hirohiko Hirano

**Affiliations:** ^1^ Department of Dentistry and Oral Surgery National Center for Geriatrics and Gerontology Obu Japan; ^2^ Research Team for Promoting Independence and Mental Health Tokyo Metropolitan Institute of Gerontology Tokyo Japan; ^3^ Gerodontology, Department of Oral Health Science, Faculty of Dental Medicine Hokkaido University Sapporo Japan; ^4^ Japan Environment and Children's Study Programme Office National Institute for Environmental Studies Ibaraki Japan; ^5^ Research Team for Social Participation and Community Health Tokyo Metropolitan Institute of Gerontology Tokyo Japan; ^6^ Research Team for Human Care Tokyo Metropolitan Institute of Gerontology Tokyo Japan; ^7^ Faculty of Health Care Takasaki University of Health and Welfare Gunma Japan; ^8^ Department of Social Medicine Hirosaki University School of Medicine Aomori Japan; ^9^ Graduate School of Nutrition and Health Science Kagawa Nutrition University Sakato Japan; ^10^ Tokyo Metropolitan Geriatric Hospital Tokyo Japan

**Keywords:** sarcopenia, Asian Working Group for Sarcopenia, oral function, oral hypofunction, older adults

## Abstract

**Background:**

Few studies have examined the state of oral function in older adults with sarcopenia. We assessed the oral functions of community‐dwelling older adults with sarcopenia from multiple perspectives to clarify their potentially low oral function.

**Methods:**

A total of 1517 (86.2%; 990 women, 527 men; mean age 76.1 ± 7.6 years) participants were included in this study. Grip strength, gait speed and skeletal muscle mass index were assessed, and sarcopenia was evaluated according to the criteria of the Asian Working Group for Sarcopenia 2019. The degree of tongue coating, oral moisture, occlusal force, tongue–lip motor function, tongue pressure, masticatory function and swallowing function were assessed. The criteria for oral hypofunction (a disease that is a combination of multiple low oral functions) were used to assess oral function. Statistical analyses were performed using Kolmogorov–Smirnov test, unpaired *t*‐test, Mann–Whitney *U* test, *χ* ^2^ test, and univariate and multivariable logistic regression analyses, with each oral function as the dependent variable and sarcopenia as one of the independent variables. The significance level was set at *P* < 0.05.

**Results:**

The prevalence rates of sarcopenia and severe sarcopenia were 14.2% and 3.8%, respectively. The prevalence of oral hypofunction was 39.9%. Compared with the robust group, the sarcopenia and severe sarcopenia groups tended to have a higher frequency of the following components (all *P* < 0.01): low occlusal force, low tongue–lip motor function, low tongue pressure, low masticatory function, low swallowing function and oral hypofunction. Univariate logistic regression analysis showed that sarcopenia was associated with low occlusal force, low tongue–lip motor function, low tongue pressure, low masticatory function, low swallowing function and oral hypofunction. The odds ratios and 95% confidence intervals of sarcopenia for each oral function were 2.62 [2.00, 3.43], 2.21 [1.69, 2.89], 3.66 [2.79, 4.81], 3.23 [2.46, 4.25], 1.66 [1.26, 2.20] and 3.59 [2.72, 4.72], respectively. Multivariable logistic regression analysis showed that sarcopenia was associated with low occlusal force (1.63 [1.10, 2.40]), low tongue pressure (2.28 [1.65, 3.15]), low masticatory function, (1.94 [1.27, 2.97]), low swallowing function (1.64 [1.17, 2.28]) and oral hypofunction (2.17 [1.52, 3.09]).

**Conclusions:**

This study demonstrated that multiple aspects of oral function were low among community‐dwelling older adults with sarcopenia. The potential decline in oral functions in older adults with sarcopenia may have been overlooked until now. This study indicates the need for dental perspectives in intervening with older adults with sarcopenia and the need to encourage them to see dental professionals.

## Introduction

Perioral muscles related to tongue pressure,^1^, ^2^, ^3^ tongue thickness,^2^, ^4^ dexterity of tongue,^2^ masseter muscle thickness,^5^ jaw‐opening force,^1^ masticatory function^6^, ^7^ and swallowing function^7^, ^8^, ^9^ have been reported to be associated with sarcopenia. Furthermore, oral hypofunction,^10^ a disease that is a combination of multiple low oral functions, is also reported to be associated with sarcopenia.^11^, ^12^, ^13^ These reports suggest a positive correlation between the maxillofacial region, systemic muscles and motor performance.

It is reported that low oral functions are associated with lower intake of several nutrients, including protein, and undernutrition,^14^, ^15^, ^16^, ^17^, ^18^ which is a factor in sarcopenia.^8^ Previously, this pathway by which low oral function leads to sarcopenia via poorer nutritional status has been examined.^6^, ^7^, ^11^, ^12^, ^13^ However, the possibility that sarcopenia may precede or coincide with low oral function cannot be ruled out, given the apparent association between perioral and systemic muscles.

If oral functions, which are potentially declining in older adults with sarcopenia, are left untreated and only nutritional intervention is used, the intervention's effectiveness may not be fully realized. Therefore, identifying potential low oral functions in older adults with sarcopenia will provide important evidence for considering different intervention methods. In this study, we hypothesized that multiple oral functions will be decreased in older adults with sarcopenia. This cross‐sectional study aimed to examine the oral functions of community‐dwelling older adults with sarcopenia from multiple perspectives.

## Methods

### Study design

This is a cross‐sectional study that uses data of participants from the Kusatsu Study and two Otassha Studies of 2018; these are cohort studies conducted by the Tokyo Metropolitan Institute of Gerontology on older adults aged 65 years and above. The Kusatsu Study was conducted in Kusatsu town in Gunma Prefecture, a rural area, and the Otassha Studies were conducted in Itabashi City, Tokyo, an urban area, among community‐dwelling older adults. The study designs of the Kusatsu Study and two Otassha Studies have been reported earlier.^19^, ^20^, ^21^, ^22^, ^23^ Across all three studies, participants were informed of the date and venue of the comprehensive health examination, by mail, in advance; a comprehensive health examination was conducted for those who appeared on the allotted date. The purpose and content of this activity were explained to all participants orally and in writing, and their written consent was obtained. Furthermore, two exclusion criteria were set: (1) participants with at least one missing endpoint for sarcopenia and (2) participants with at least one missing endpoint for oral hypofunction. All evaluators in each study received training on measurement methods and how to use evaluation instruments in advance, and the evaluation criteria were unified. This study was conducted with the approval of the Ethics Committee of the Tokyo Metropolitan Institute of Gerontology (2006‐17, 2008‐3, 2011‐48, 2018‐Zin1, 15, 16) and was designed in accordance with the Strengthening the Reporting of Observational Studies in Epidemiology Statement.

### Sarcopenia

To determine sarcopenia, the grip strength, gait speed, and appendicular skeletal muscle mass of the participants were measured. Referring to the Asian Working Group for Sarcopenia 2019 (AWGS2019), sarcopenia was characterized by low appendicular skeletal muscle mass and low muscle strength or low physical performance, and severe sarcopenia was characterized by all the three conditions being present.^24^ The survey items used to determine sarcopenia were assessed by researchers who had expertise in motor function.

#### Low muscle strength

To evaluate muscle strength, the grip strength of the dominant hand was measured using a Smedley‐type hand dynamometer (Grip‐A, Takei Scientific Instruments Co., Ltd., Niigata, Japan). The participants had to hold the grip of the hand dynamometer in an upright posture with their arms naturally lowered, and the angle of bending the second joint of the index finger was adjusted to 90°. The measurements were taken twice, and the higher value was taken as the grip strength. Grip strength of <28 kg for men and <18 kg for women was defined as low muscle strength.^24^

#### Low physical performance

Gait speed was measured to evaluate physical performance. The participants were instructed to walk at a normal pace along an 11‐m straight sidewalk with 3‐ and 8‐m points marked with tape on a flat floor. The time taken to walk a 5‐m distance between the tapes was measured, and the gait speed was calculated. A gait speed of <1.0 m/s was defined as low physical performance.^24^

#### Low appendicular skeletal muscle mass

The appendicular skeletal muscle mass was measured using bioelectrical impedance analysis (InBody 720, InBody Inc., Seoul, Korea); it was divided by the square of the height (in metres) to calculate the skeletal muscle mass index (SMI). An SMI of <7.0 kg/m^2^ for men and <5.7 kg/m^2^ for women was defined as low appendicular skeletal muscle mass.^24^

### Oral functions

Oral functions were assessed by investigating the following seven items: the degree of tongue coating, oral moisture, occlusal force, tongue–lip motor function, tongue pressure, masticatory function and swallowing function. According to the criteria for oral hypofunction, participants were evaluated for the presence of poor oral hygiene, oral dryness, low occlusal force, low tongue–lip motor function, low tongue pressure, low masticatory function and low swallowing function.^10^ Those who had three or more of the above seven functional declines were regarded to have oral hypofunction.^10^ Those who regularly used removable dentures participated in oral function tests while wearing them. Oral function assessments were performed in the following order: tongue–lip motor function, oral moisture, the degree of tongue coating, occlusal force, masticatory function, tongue pressure and swallowing function. Oral‐related assessments were performed by dentists and dental hygienists.

#### Poor oral hygiene

The degree of tongue coating was evaluated using the tongue coating index (TCI). The dorsum of the tongue was divided into nine blocks, and the degree of tongue coating on each block was visually inspected on a scale of 0–2.^25^ The total score of the nine blocks was calculated as the percentage of TCI; a TCI of ≥50% was defined as poor oral hygiene.^10^

#### Oral dryness

The degree of oral moisture was evaluated using an oral moisture checker (Mucus, Life Co., Ltd., Saitama, Japan).^26^ The 7.2‐mm^2^ sensor at the tip of the device was covered with a disposable cover and pressed against the central area of the tongue dorsum at approximately 200 gf to measure the moisture content of the tongue.^26^ The measurements were taken three times in succession, and the median value was used. Oral dryness was defined as an oral moisture level of <27.0.^10^

#### Low occlusal force

The occlusal force was measured using a pressure‐sensitive sheet (Dental Prescale 50H Type‐R, Fuji Film Co., Tokyo, Japan) and analysed using an image scanner (Occluzer, FPD‐707, Fuji Film Co.).^27^ A horseshoe‐shaped–pressure‐sensitive sheet with a thickness of 98 μm was inserted into the oral cavity to cover the entire dentition; the participant was made to clench with maximum force for approximately 3 s while in the maximal intercuspal position.^27^ The contact points of the upper and lower teeth or dental prosthesis marked on the pressure‐sensitive sheet were read by an image scanner, and the occlusal force was calculated from the area and strength of the contact pressure. An occlusal force of <200 N was defined as a low occlusal force.^10^

#### Low tongue–lip motor function

For this function, the dexterity of the tongue was measured using an oral function measuring device (KENKOU‐KUN handy, Takei Scientific Instruments Co., Ltd.).^28^ The /ta/ sound, which is one of the pronunciations used to evaluate tongue and lip motor function, was repeatedly made as quickly as possible for 5 seconds into the device's microphone, and the number of syllables pronounced per second was evaluated.^28^ The number of /ta/ pronunciations per second was defined as the oral diadochokinesis (ODK) /ta/, and if the number was <6, it was defined as low tongue–lip motor function.^10^

#### Low tongue pressure

Tongue pressure was assessed using a tongue pressure measurement device (JMS tongue pressure device TPM‐01; JMS Co., Ltd., Hiroshima, Japan).^29^ The balloon of the disposable oral probe was inflated with 19.6‐kP air to a diameter of approximately 18 mm and a volume of 3.7 mL, and the pressure was corrected to zero before measurement. The inflated balloon was placed in the anterior part of the palate and pressed against the palate with the tongue with maximum force for approximately 7 s.^29^ The measurements were taken thrice, and the average of the maximum pressures recorded was used. A tongue pressure of <30 kPa was defined as low tongue pressure.^10^

#### Low masticatory function

Masticatory function was evaluated using gummy jelly (the test gummy jelly, UHA Mikakuto Co., Ltd., Osaka, Japan)^30^; 5.50 ± 0.05 g of gummy jelly was chewed 30 times and then discharged into gauze. The masticatory function was evaluated by visually comparing the gummy jelly crushed via chewing with a score chart on a scale of 10 from 0 to 9.^30^ A score of ≤2 was defined as a low masticatory function.^10^

#### Low swallowing function

Swallowing function was evaluated using the 10‐item Eating Assessment Tool (EAT‐10), a self‐administered questionnaire that has participants answer 10 questions about their swallowing function on a 5‐point Likert scale from 0 to 4.^31^ A total score of ≥3 was defined as a low swallowing function.^10^

### Other recorded variables

Other survey items included the number of present teeth and functional teeth,^12^ age, years of education, body mass index, drinking and smoking habits, and living situation. Furthermore, the following surveys were also employed: Japan Science and Technology Agency Index of Competence^32^ for higher level functional capacity; Mini‐Mental State Examination^33^ for cognitive function, medical history obtained via interview (heart disease, diabetes and stroke); and blood tests for serum albumin and haemoglobin A1c.

### Statistical methods

Participants without sarcopenia were included in the robust group. As the number of participants with severe sarcopenia was not sufficient for analysis (57 [3.8%]), we did not distinguish between sarcopenia and severe sarcopenia and included them in the sarcopenia group itself. The normality of continuous variables was checked using the Kolmogorov–Smirnov test. Continuous variables with a normal distribution were compared between the two groups using an unpaired *t*‐test, and those with a non‐normal distribution were compared using the Mann–Whitney *U* test. Intergroup comparisons of categorical variables were performed using the *χ* ^2^ test. The association between sarcopenia and low oral function was examined using multivariable logistic regression analysis, with each low oral function as the dependent variable and the presence of sarcopenia as the independent variable. The correlation coefficients between independent variables entered in the multivariable logistic regression were evaluated using Spearman's rank correlation coefficient (*Supporting Information Table* *S1*). The results showed that the largest correlation coefficient was 0.53 for gender and smoking, followed by 0.49 for diabetes and HbA1c. The low correlation coefficients among the independent variables indicated that multicollinearity was unlikely to occur among them. Statistical analysis was performed using IBM SPSS version 28 (IBM Corp., Armonk, NY, USA). As this study is secondary to the Kusatsu Study and each Otassha Study, no prior sample size calculations were performed.

## Results

### Participants

**Figure 1:**
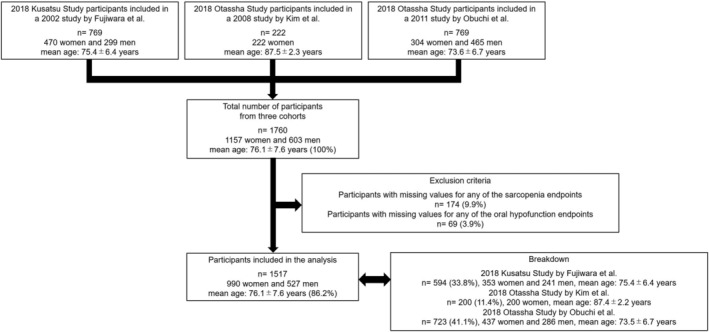
Flowchart of this study. The total number of participants in the three cohort studies in 2018 was 1760. After excluding those who met the exclusion criteria, 1517 participants were included in the analysis.

A flowchart of the study is demonstrated in *Figure* *1*. The total number of participants across the three studies in 2018 was 1760 (100%; 1157 women and 603 men, mean age 76.1 ± 7.6 years). In total, 243 (13.8%) participants were excluded as they met either of the two predefined exclusion criteria: 174 (9.9%) with missing values for any of the sarcopenia endpoints and 69 (3.9%) with missing values for any of the oral hypofunction endpoints. Thus, the final number of participants was 1517 (86.2%; 990 women and 527 men; mean age 76.1 ± 7.6 years).

### Descriptive datum

*Table* *1* shows the characteristics of the study population. *Table* *2* shows the details of the variables on sarcopenia and oral hypofunction in the participants. The overall prevalence of sarcopenia and severe sarcopenia according to AWGS2019 criteria were 14.2% (*n* = 216) and 3.8% (*n* = 57), respectively, with 16.6% (*n* = 164) and 4.8% (*n* = 48) among women, and 9.9% (*n* = 52) and 1.7% (*n* = 9) among men, respectively. The prevalence of low muscle strength, physical performance and appendicular skeletal muscle mass, according to the AWGS2019 criteria, were 25.1%, 12.0% and 38.4%, respectively. The prevalence of oral hypofunction was 39.9% (*n* = 605) overall: 43.6% (*n* = 432) among women and 32.8% (*n* = 173) among men, and was 34.3% (*n* = 427) and 65.2% (*n* = 178) in the robust and sarcopenia groups (a group that includes sarcopenia and severe sarcopenia), respectively. These results demonstrate a significant difference between the two groups. Among the oral functions, occlusal force, oral diadochokinesis /ta/, tongue pressure, gummy jelly score, EAT‐10 score and oral hypofunction scores were significantly different between the robust and sarcopenia groups. The prevalence of the following low oral functions was significantly higher in the sarcopenia group than in the robust group: low occlusal force, low tongue–lip motor function, low tongue pressure, low masticatory function, low swallowing function and oral hypofunction.

**Table 1:** Characteristics of the participants of this study

		Overall (*n* = 1517)	*P* value	Robust (*n* = 1244)	Sarcopenia (*n* = 273)	*P* value
Characteristics		Median (Q1, Q3)/*N* (%)		Median (Q1, Q3)/*N* (%)	Median (Q1, Q3)/*N* (%)	
Age (years)		76 (69, 82)	<0.01^a^	74 (68, 80)	84 (77, 88)	<0.01^b^
Sex	Women	990 (65.3)		778 (62.5)	212 (77.7)	<0.01^c^
Number of present teeth		22 (11, 26)	<0.01^a^	23 (12, 27)	16 (4, 24)	<0.01^b^
	<20	647 (42.6)		497 (40.0)	150 (54.9)	<0.01^c^
Number of functional teeth		28 (27, 28)	<0.01^a^	28 (27, 28)	28 (27, 28)	0.17^b^
Body mass index (kg/m^2^)		22.6 (20.6, 24.8)	<0.01^a^	23.0 (21.1, 25.2)	20.8 (18.8, 22.7)	<0.01^b^
	<18.5	136 (9.0)		77 (6.2)	59 (21.6)	<0.01^c^
Education (years)		12 (9, 14)	<0.01^a^	12 (10, 14)	11 (9, 12)	<0.01^b^
Lifestyle habits						
Daily drinking habit		282 (18.6)		248 (19.9)	34 (12.5)	<0.01^c^
Smoking habit	Never Smoked	1,002 (66.1)		801 (64.4)	201 (73.9)	<0.01^c^
	Used to smoke	392 (25.9)		336 (27.0)	56 (20.6)	
	Smoking	122 (8.0)		107 (8.6)	15 (5.5)	
Living situation	Living alone	464 (30.8)		362 (29.3)	102 (37.6)	<0.01^c^
Functional capacities						
JST‐IC score		12 (9, 14)	<0.01^a^	12 (10, 14)	9 (7, 12)	<0.01^b^
MMSE score		29 (28, 30)	<0.01^a^	29 (28, 30)	29 (27, 29)	<0.01^b^
	28≤	1,089 (78.2)		921 (80.6)	168 (66.9)	<0.01^c^
	24–27	250 (17.9)		195 (17.1)	55 (21.9)	
	≤23	54 (3.9)		26 (2.3)	28 (11.2)	
Comorbidities						
Heart disease		231 (15.2)		182 (14.6)	49 (18.0)	0.16^c^
Diabetes		192 (12.7)		159 (12.8)	33 (12.1)	0.77^c^
Stroke		84 (5.5)		73 (5.9)	11 (4.0)	0.23^c^
Blood tests						
Serum albumin (g/dL)		4.2 (4.1, 4.4)	<0.01^a^	4.2 (4.1, 4.4)	4.2 (4.0, 4.3)	<0.01^b^
Haemoglobin A1c (%)		5.7 (5.5, 6.0)	<0.01^a^	5.7 (5.5, 6.0)	5.6 (5.4, 6.0)	0.23^b^

Abbreviations: Q1, first quartile; Q3, third quartile; EAT, Eating Assessment Tool; JST‐IC, Japan Science and Technology Agency Index of Competence; MMSE, Mini‐Mental State Examination.

Continuous variables showed a non‐normal distribution through the Kolmogorov–Smirnov test; hence, they were described with the median (first quartile, third quartile) and analysed using the Mann–Whitney *U* test. Categorical variables were statistically analysed using the *χ* ^2^ test.

^a^ Kolmogorov–Smirnov test.

^b^ Mann–Whitney *U* test.

^c^ *χ* ^2^ test.

**Table 2:** Variables on sarcopenia and oral hypofunction in the participants

		Overall (*n* = 1517)	*P* value	Robust (*n* = 1244)	Sarcopenia (*n* = 273)	*P* value
Continuous variables on sarcopenia		Median (Q1, Q3)		Median (Q1, Q3)	Median (Q1, Q3)	
Handgrip strength (kg)		24.0 (19.0, 31.0)	<0.01^a^	25.0 (20.5, 33.0)	16.0 (14.0, 20.3)	<0.01^c^
Gait speed (m/s)		1.3 (1.1, 1.5)	<0.01^a^	1.4 (1.2, 1.5)	1.1 (0.9, 1.3)	<0.01^c^
Skeletal muscle mass index (kg/m^2^)		6.2 (5.6, 7.1)	<0.01^a^	6.4 (5.9, 7.3)	5.3 (4.9, 5.7)	<0.01^c^
Categorical variables on sarcopenia		N (%)		N (%)	N (%)	
Low muscle strength		381 (25.1)		146 (11.7)	235 (86.1)	<0.01^d^
Low physical performance		182 (12.0)		87 (7.0)	95 (34.8)	<0.01^d^
Low appendicular skeletal muscle mass		583 (38.4)		310 (24.9)	273 (100)	<0.01^d^
Sarcopenia	Robust	1244 (82.0)		1244 (100)	0 (0)	<0.01^d^
	sarcopenia	216 (14.2)		0 (0)	216, (79.1)	
	Severe sarcopenia	57 (3.8)		0 (0)	57 (20.9)	
Continuous variables on oral hypofunction		median (Q1, Q3)/mean ± *SD*		median (Q1, Q3)/mean ± *SD*	median (Q1, Q3)/mean ± *SD*	
Tongue coating index (%)		16.7 (5.6, 50.0)	<0.01^a^	16.7 (5.6, 50.0)	16.7 (8.3, 38.9)	0.18^c^
Oral moisture		27.7 (25.7, 29.3)	<0.01^a^	27.7 (25.8, 29.3)	27.3 (25.4, 29.2)	0.14^c^
Occlusal force (N)		246.4 (116.6, 400.9)	<0.01^a^	272.8 (134.9, 421.1)	153.7 (61.2, 284.1)	<0.01^c^
Oral diadochokinesis /ta/ (time/s)		6.2 (5.8, 6.8)	<0.01^a^	6.4 (5.8, 6.8)	6.0 (5.4, 6.4)	<0.01^c^
Tongue pressure (kPa)		30.5 ± 8.1	0.05^a^	31.6 ± 7.8	25.5 ± 7.2	<0.01^b^
Gummy jelly score		5 (2, 6)	<0.01^a^	5 (3, 6)	3 (0.5, 6)	<0.01^c^
EAT‐10 score		1 (0, 3)	<0.01^a^	0.5 (0, 3)	1 (0, 5)	<0.01^c^
Oral hypofunction score		2 (1, 3)	<0.01^a^	2 (1, 3)	3 (2, 4)	<0.01^c^
Categorical variables on oral hypofunction		*N* (%)		*N* (%)	*N* (%)	
Poor oral hygiene		403 (26.6)		343 (27.6)	60 (22.0)	0.06^d^
Oral dryness		590 (38.9)		471 (37.9)	119 (43.6)	0.08^d^
Low occlusal force		640 (42.2)		472 (37.9)	168 (61.5)	<0.01^d^
Low tongue–lip motor function		446 (29.4)		326 (26.2)	120 (44.0)	<0.01^d^
Low tongue pressure		567 (37.4)		395 (31.8)	172 (63.0)	<0.01^d^
Low masticatory function		391 (25.8)		264 (21.2)	127 (46.5)	<0.01^d^
Low swallowing function		416 (27.4)		317 (25.5)	99 (36.3)	<0.01^d^
Oral hypofunction		605 (39.9)		427 (34.3)	178 (65.2)	<0.01^d^

Abbreviations: Q1, first quartile; Q3, third quartile; *SD*, standard deviation; EAT, Eating Assessment Tool.

Tongue pressures that showed a normal distribution using the Kolmogorov–Smirnov test were described by the mean ± standard deviation and analysed using the unpaired *t*‐test. Other continuous variables that showed non–normal distribution by the Kolmogorov–Smirnov test were described with the median (first quartile, third quartile) and analysed using the Mann–Whitney *U* test. Categorical variables were statistically analysed using the chi‐square test.

^a^ Kolmogorov–Smirnov test.

^b^ Unpaired *t*‐test.

^c^ Mann–Whitney *U* test.

^d^ *χ* ^2^ test.

### Results of statistical analyses

The results of the univariate and multivariable logistic regression analyses, demonstrating the presence or absence of low oral function as the dependent variable and that of sarcopenia as the independent variable, are shown in *Table* *3*. The results showed that low occlusal force, low tongue–lip motor function, low tongue pressure, low masticatory function, low swallowing function and oral hypofunction were significantly associated with sarcopenia. The results of multivariable logistic regression analysis, after adjusting for the effects of multiple independent variables, showed that the following low oral functions were significantly associated with sarcopenia: low occlusal force, low tongue pressure, low masticatory function, low swallowing function and oral hypofunction. The results of each independent variable in the multivariable logistic regression analysis with the presence or absence of each low oral function as the dependent variable are described in the *Tables* *S2–S9*.

**Table 3:** Association between low oral function and sarcopenia using the univariate and multivariable logistic analysis

Crude model		95% Confidence intervals	
Dependent variables	Odds ratio	Lower limit	Upper limit
Poor oral hygiene	0.74	0.54	1.01
Oral dryness	1.27	0.97	1.65
**Low occlusal force**	**2.62**	**2.00**	**3.43**
**Low tongue–lip motor function**	**2.21**	**1.69**	**2.89**
**Low tongue pressure**	**3.66**	**2.79**	**4.81**
**Low masticatory function**	**3.23**	**2.46**	**4.25**
**Low swallowing function**	**1.66**	**1.26**	**2.20**
**Oral hypofunction**	**3.59**	**2.72**	**4.72**

Adjusted model			
Poor oral hygiene	0.81	0.56	1.17
Oral dryness	1.10	0.80	1.52
**Reduced occlusal force**	**1.63**	**1.10**	**2.40**
Decreased tongue–lip motor function	1.28	0.92	1.78
**Decreased tongue pressure**	**2.28**	**1.65**	**3.15**
**Decreased masticatory function**	**1.94**	**1.27**	**2.97**
**Deterioration of swallowing function**	**1.64**	**1.17**	**2.28**
**Oral hypofunction**	**2.17**	**1.52**	**3.09**

Abbreviations: JST‐IC, Japan Science and Technology Agency Index of Competence; MMSE, Mini‐Mental State Examination.

*Note*: The results of univariate and multivariable logistic analysis with low oral function as the dependent variable and the presence of sarcopenia as the independent variable are presented. Odds ratios and 95% confidence intervals for sarcopenia, along with the independent variables in each univariate and multivariable logistic analyses, were described. Low oral function that was significantly associated with sarcopenia is shown in bold.

Independent variables other than sarcopenia (0: Robust, 1: Sarcopenia) include age, sex (0:Women, 1:Men), number of present teeth, daily drinking habits (0: No, 1: Yes), smoking habit (0: Never smoked, 1: Used to smoke, 2: Smoking), living situation (0: Living with someone, 1: Living alone), education, JST‐IC score, MMSE score, heart disease (0: No, 1: Yes), diabetes (0: No, 1: Yes), stroke (0: No, 1: Yes), serum albumin, haemoglobin A1c, cohort studies (1: Kusatsu Study, 2: Otassha Study by Kim et al., 3: Otassha Study by Obuchi et al.)

## Discussion

Intervention methods for sarcopenia are currently being investigated from multiple perspectives. One of these interventions is nutritional intervention, although oral function has rarely been examined in previous nutritional interventions. Older adults with sarcopenia may have decreased oral functions as their muscle mass declines systematically. In order to increase perspectives on intervention points for sarcopenia, this study was conducted to assess oral functions in older adults with sarcopenia from multiple perspectives to identify potentially low oral functions.

The prevalence of sarcopenia and severe sarcopenia in the combined data from two regions with different characteristics, Kusatsu‐cho (rural area) and Itabashi Ward (urban area), was 13.1% and 3.3%, respectively. Additionally, the prevalence of sarcopenia and severe sarcopenia for the AWGS2019 criteria in Asian community‐dwelling older adults have been reported to be 8.8%–22.8% and 1.2%–13.7%, respectively.^34^, ^35^, ^36^, ^37^ The participants in this study were within the range of prevalence reported in recent academic literature. In the present study, the prevalence of oral hypofunction was 38.4%. Additionally, the incidence of oral hypofunction among older adults living in other regions has been reported to be 35.9%–63.0%.^11^, ^13^, ^38^, ^39^, ^40^ The prevalence of oral hypofunction was within the range reported previously, as were the prevalence of sarcopenia and oral hypofunction. Those results were obtained by integrating data from rural (39.2%) and urban (60.8%) areas to reduce regional bias. Additionally, to further reduce regional bias and generalize the results of this study, each cohort was adjusted as an adjustment variable in the multivariable logistic regression analysis.

In the intergroup comparison of oral function between the robust and sarcopenia groups, several oral functions were significantly lower in the sarcopenia group. The results of multivariable logistic regression analysis, with each oral function as the dependent variable, showed that low occlusal force, low tongue pressure, low masticatory function, low swallowing function and oral hypofunction were significantly associated with sarcopenia. This study revealed that older adults with sarcopenia have low oral functions, whereas poor oral hygiene and oral dryness were not associated with sarcopenia. Similar results have been reported in a study of older adults living in other regions.^11^, ^13^ Tongue coating is reported to be strongly influenced by oral hygiene.^41^ Additionally, multiple factors have shown to influence xerostomia, a condition in which saliva secretion is reduced, and the mouth is dry, including endocrinologic causes, such as diabetes mellitus and autoimmune thyroid, and medications, such as anticholinergic agents.^42^ It is likely that factors other than sarcopenia were more strongly related to the decline in these two functions. Additionally, in the present study, no association was found between low tongue–lip motor function and sarcopenia in the multivariable logistic regression analysis. This is consistent with findings from other studies.^11^ It has been reported that the pronunciation function is maintained to some extent even in older adults who require long‐term care^43^ and that the size and relative frequency of fast myosin muscle fibres of the styloglossus, one of the muscles that make up the tongue, is preserved with old age and may be resistant to sarcopenia [S10]. These results suggest that the decrease in tongue and lip movements related to pronunciation are unlikely to be affected by the decline in appendicular skeletal muscle mass, muscle strength and physical performance. However, the relationship between perioral muscle mass, such as masseter and tongue muscles, and systemic muscles has already been reported.^1^, ^2^, ^3^, ^4^, ^5^, ^8^, ^9^ Furthermore, the association between the deterioration of swallowing function and sarcopenia has been reported in several studies.^3^, ^7^, ^8^, ^9^ Based on the results of this study and previous reports, it can be concluded that muscle mass, muscle strength, physical performance of the whole body, and the muscle mass and muscle strength of the perioral muscles are not independent of each other.

Older adults with poor oral function tended to avoid hard foods such as meats, fruits and vegetables, which are typically major sources of proteins, fibre, vitamins and minerals. Low protein intake has been associated with considerable loss of muscle mass and strength (S11–S13). Low masticatory function has been shown to be detrimental to systemic metabolism of ingested protein (S14). In this study, sarcopenia showed high odds ratios not only for individual oral functions but also for oral hypofunction. These findings indicate that nutritional interventions that promote protein intake, which is important for skeletal muscle, may not be maximally effective if implemented while oral health problems are left unchecked. Simultaneous prosthetic treatment and simple dietary advice have been reported to possibly improve both masticatory function and nutritional status (S15, S16). Appropriate treatment of oral health problems by dental professionals may contribute to increasing the effectiveness of nutritional interventions, such as those for sarcopenia.

The novel findings of this study indicate that a single oral function assessment of older adults with sarcopenia may miss the low oral function and miss the appropriate intervention point. One effective tool for nondental professionals is to first perform a brief screening for low oral function in older adults with sarcopenia. Then, if necessary, leading towards thorough examinations by dental professionals could result in appropriate oral health care.

Recently, the concept of oral frailty has been introduced in Japan.^7^, ^20^ A longitudinal study has reported that oral frailty, a state of duplication of poor oral function, leads to physical frailty, sarcopenia, disability and mortality.^7^ Habitual inadequate food intake, worsened nutritional status and other factors are considered to mediate the relationship between oral frailty and adverse events. However, it is unlikely that the decline in oral function, such as oral frailty, precedes sarcopenia in all older adults with sarcopenia, and it cannot be denied that there are cases where sarcopenia precedes and leads to a decline in oral function.^9^ As the muscle mass and muscle strength of the perioral muscles are related to the systemic muscles,^1^, ^2^, ^3^, ^4^, ^5^, ^8^, ^9^ it is thought that there is also a pathway in which the muscle mass and muscle strength of perioral muscles decrease along with a decrease in the muscle mass, muscle strength and physical performance of the whole body. However, as this was a cross‐sectional study, the pathway could not be proven. This is the limitation of the present study. In the future, it will be necessary to examine the relationship between sarcopenia and oral function longitudinally.

In this study, we report the details of a multidimensional evaluation of oral function in older adults with sarcopenia. In this sample group, the tongue strength, which is the largest organ in the oral cavity, the power to bite food and the masticatory and swallowing functions that integrate those functions decreased; furthermore, these low oral functions were found to be overlapping. This study revealed that older adults with sarcopenia have potentially low oral functions. The study also showed that sarcopenia might be one of the factors that lead to low oral functions, which are largely influenced by muscle strength and muscle mass in the maxillofacial region. Therefore, early intervention by dental professionals for low oral function may be necessary to prevent the decline in nutritional status and severity of sarcopenia caused by the decrease in oral function expected to occur in the future across older adults with sarcopenia.

## Conclusions

The following oral functions were found to be low in community‐dwelling older adults with sarcopenia: occlusal force, tongue–lip motor function, tongue pressure, masticatory function and swallowing function. It was also observed that these low functions overlapped in older adults with sarcopenia. A multivariable logistic regression analysis showed that sarcopenia was associated with low occlusal force, low tongue pressure, low masticatory function, low swallowing function and oral hypofunction. Consequently, sarcopenia may be a factor in the decline of oral function. Intervening with the older adults with sarcopenia from dental perspectives as well, and encouraging them to see dental professionals when necessary, may free them from the negative cycle of sarcopenia and low oral function.

## Conflict of interest

The authors declare no conflict of interest. The funders had no role in the design of the study; collection, analyses or interpretation of data; writing of the manuscript; or the decision to publish the results.

## Supporting information


**Table S1.** Spearman's rank correlation coefficient of independent variables
**Table S2.** Association between poor oral hygiene and sarcopenia
**Table S3.** Association between oral dryness and sarcopenia
**Table S4.** Association between low occlusal force and sarcopenia
**Table S5.** Association between low tongue–lip motor function and sarcopenia
**Table S6.** Association between low tongue pressure and sarcopenia
**Table S7.** Association between low masticatory function and sarcopenia
**Table S8.** Association between low of swallowing function and sarcopenia
**Table S9.** Association between oral hypofunction and sarcopenia
**Supporting Information.** References S10 to S16
