# Supplementary material for: Association between sarcopenia and oral functions in community‐dwelling older adults: A cross‐sectional study
Source: J Cachexia Sarcopenia Muscle. 2022 Dec 5;14(1):429–38. doi: 10.1002/jcsm.13145 (PMC9891969; doi:10.1002/jcsm.13145)
Supplement: Supplementary file 1 — Table S1. Spearman's rank correlation coefficient of independent variables Table S2. Association between poor oral hygiene and sarcopenia Table S3. Association between oral dryness and sarcopenia Table S4. Association between low occlusal force and sarcopenia Table S5. Association between low tongue–lip motor function and sarcopenia Table S6. Association between low tongue pressure and sarcopenia Table S7. Association between low masticatory function and sarcopenia Table S8. Association between low of swallowing function and sarcopenia Table S9. Association between oral hypofunction and sarcopenia Supporting Information. References S10 to S16 [file JCSM-14-429-s001.docx]

**Association Between Sarcopenia and Oral Functions in Community-Dwelling Older Adults: A Cross-Sectional Study**

Yoshihiro Kugimiya^1*^, Masanori Iwasaki^2^, Yuki Ohara^2^, Keiko Motokawa^2^, Ayako Edahiro^2^, Maki Shirobe^2^, Yutaka Watanabe^2,3^, Yu Taniguchi^4^, Satoshi Seino^5^, Takumi Abe^5^, Shuichi Obuchi^6^, Hisashi Kawai^6^, Takeshi Kera^6,7^, Yoshinori Fujiwara^5^, Akihiko Kitamura^5^, Kazushige Ihara^8^, Hunkyung Kim^2^, Shoji Shinkai^5,9^, Hirohiko Hirano^2,10^

1. Department of Dentistry and Oral Surgery, National Center for Geriatrics and Gerontology, Obu, Japan

2. Research Team for Promoting Independence and Mental Health, Tokyo Metropolitan Institute of Gerontology, Tokyo, Japan

3. Gerodontology, Department of Oral Health Science, Faculty of Dental Medicine, Hokkaido University, Sapporo, Japan

4. Japan Environment and Children’s Study Programme Office, National Institute for Environmental Studies, Ibaraki, Japan

5. Research Team for Social Participation and Community Health, Tokyo Metropolitan Institute of Gerontology, Tokyo, Japan

6. Research Team for Human Care, Tokyo Metropolitan Institute of Gerontology, Tokyo, Japan

7. Faculty of Health Care, Takasaki University of Health and Welfare, Gunma, Japan

8. Department of Social Medicine, Hirosaki University School of Medicine, Aomori, Japan

9. Graduate School of Nutrition and Health Science, Kagawa Nutrition University, Sakato, Japan

10. Tokyo Metropolitan Geriatric Hospital, Tokyo, Japan

***Corresponding author**: Yoshihiro Kugimiya

National Center for Geriatrics and Gerontology,

7-430 Morioka-cho, Obu City, Aichi Prefecture 474-8511, Japan

E-mail: kugimiyay@ncgg.go.jp

[Phone number] +81-562-46-2311

[Fax number] +81-562-44-8518

Supporting Information Table S1: Spearman's rank correlation coefficient of independent variables

|  |  | Sarcopenia | Age | Sex | Number of present teeth | Daily drinking habits | Smoking habit | Living situation | Education | JST-IC | MMSE | Heart disease | Diabetes | Stroke | Serum albumin | Hemoglobin A1c | Cohort studies |
| --- | --- | --- | --- | --- | --- | --- | --- | --- | --- | --- | --- | --- | --- | --- | --- | --- | --- |
| Sarcopenia | CC | 1.00 |  |  |  |  |  |  |  |  |  |  |  |  |  |  |  |
|  | p-value |  |  |  |  |  |  |  |  |  |  |  |  |  |  |  |  |
| Age | CC | 0.36 | 1.00 |  |  |  |  |  |  |  |  |  |  |  |  |  |  |
|  | p-value | <0.01 |  |  |  |  |  |  |  |  |  |  |  |  |  |  |  |
| Sex | CC | -0.12 | -0.20 | 1.00 |  |  |  |  |  |  |  |  |  |  |  |  |  |
|  | p-value | <0.01 | <0.01 |  |  |  |  |  |  |  |  |  |  |  |  |  |  |
| Number of present teeth | CC | -0.17 | -0.38 | 0.12 | 1.00 |  |  |  |  |  |  |  |  |  |  |  |  |
|  | p-value | <0.01 | <0.01 | <0.01 |  |  |  |  |  |  |  |  |  |  |  |  |  |
| Daily drinking habits | CC | -0.07 | -0.13 | 0.36 | 0.05 | 1.00 |  |  |  |  |  |  |  |  |  |  |  |
|  | p-value | <0.01 | <0.01 | <0.01 | 0.04 |  |  |  |  |  |  |  |  |  |  |  |  |
| Smoking habit | CC | -0.08 | -0.21 | 0.53 | -0.01 | 0.32 | 1.00 |  |  |  |  |  |  |  |  |  |  |
|  | p-value | <0.01 | <0.01 | <0.01 | 0.59 | <0.01 |  |  |  |  |  |  |  |  |  |  |  |
| Living situation | CC | 0.07 | 0.14 | -0.19 | -0.06 | -0.12 | -0.07 | 1.00 |  |  |  |  |  |  |  |  |  |
|  | p-value | <0.01 | <0.01 | <0.01 | 0.01 | <0.01 | <0.01 |  |  |  |  |  |  |  |  |  |  |
| Education | CC | -0.21 | -0.38 | 0.20 | 0.33 | 0.09 | 0.09 | -0.05 | 1.00 |  |  |  |  |  |  |  |  |
|  | p-value | <0.01 | <0.01 | <0.01 | <0.01 | <0.01 | <0.01 | 0.05 |  |  |  |  |  |  |  |  |  |
| JST-IC | CC | -0.27 | -0.32 | 0.07 | 0.20 | 0.07 | 0.00 | -0.15 | 0.32 | 1.00 |  |  |  |  |  |  |  |
|  | p-value | <0.01 | <0.01 | <0.01 | <0.01 | <0.01 | 0.90 | <0.01 | <0.01 |  |  |  |  |  |  |  |  |
| MMSE | CC | -0.18 | -0.24 | -0.02 | 0.18 | 0.02 | 0.00 | -0.01 | 0.19 | 0.27 | 1.00 |  |  |  |  |  |  |
|  | p-value | <0.01 | <0.01 | 0.47 | <0.01 | 0.54 | 0.89 | 0.61 | <0.01 | <0.01 |  |  |  |  |  |  |  |
| Heart disease | CC | 0.04 | 0.10 | 0.02 | 0.00 | 0.00 | 0.03 | 0.05 | -0.03 | -0.05 | -0.03 | 1.00 |  |  |  |  |  |
|  | p-value | 0.16 | <0.01 | 0.40 | 0.92 | 0.85 | 0.20 | 0.08 | 0.28 | 0.07 | 0.20 |  |  |  |  |  |  |
| Diabetes | CC | -0.01 | 0.03 | 0.08 | -0.04 | -0.03 | 0.05 | 0.03 | -0.05 | -0.04 | -0.05 | 0.06 | 1.00 |  |  |  |  |
|  | p-value | 0.77 | 0.27 | <0.01 | 0.15 | 0.26 | 0.07 | 0.27 | 0.08 | 0.11 | 0.05 | 0.02 |  |  |  |  |  |
| Stroke | CC | -0.03 | 0.05 | 0.08 | -0.05 | 0.00 | 0.10 | 0.02 | -0.02 | -0.08 | -0.04 | 0.08 | 0.06 | 1.00 |  |  |  |
|  | p-value | 0.23 | 0.07 | <0.01 | 0.07 | 0.92 | <0.01 | 0.39 | 0.42 | <0.01 | 0.11 | <0.01 | 0.03 |  |  |  |  |
| Serum albumin | CC | -0.11 | -0.21 | -0.05 | 0.11 | -0.07 | -0.02 | 0.00 | 0.10 | 0.07 | 0.12 | -0.01 | 0.04 | -0.01 | 1.00 |  |  |
|  | p-value | <0.01 | <0.01 | 0.05 | <0.01 | <0.01 | 0.42 | 0.93 | <0.01 | <0.01 | <0.01 | 0.81 | 0.15 | 0.64 |  |  |  |
| Hemoglobin A1c | CC | -0.03 | 0.06 | -0.02 | -0.04 | -0.07 | -0.03 | 0.02 | -0.04 | -0.02 | -0.01 | 0.03 | 0.49 | 0.05 | 0.06 | 1.00 |  |
|  | p-value | 0.23 | 0.02 | 0.48 | 0.09 | <0.01 | 0.30 | 0.54 | 0.15 | 0.45 | 0.70 | 0.18 | <0.01 | 0.04 | 0.02 |  |  |
| Cohort studies | CC | -0.07 | -0.16 | 0.01 | 0.28 | -0.04 | -0.03 | -0.06 | 0.38 | 0.18 | -0.03 | 0.02 | -0.05 | 0.01 | 0.18 | -0.03 | 1.00 |
|  | p-value | <0.01 | <0.01 | 0.75 | <0.01 | 0.12 | 0.25 | 0.02 | <0.01 | <0.01 | 0.26 | 0.47 | 0.08 | 0.62 | <0.01 | 0.24 |  |

**Abbreviations:** CC, Correlation Coefficient; JST-IC, Japan Science and Technology Agency Index of Competence; MMSE, Mini-Mental State Examination.

Supporting Information Table S2: Association between poor oral hygiene and sarcopenia

|  |  |  | 95% confidence intervals | |
| --- | --- | --- | --- | --- |
| Independent variables |  | Odds ratio | Lower limit | Upper limit |
| Sarcopenia | 0:Robust, 1:Sarcopenia | 0.81 | 0.56 | 1.17 |
| Age | 1-year increments | 1.01 | 0.99 | 1.04 |
| **Sex** | **0:Women, 1:Men** | **1.70** | **1.23** | **2.37** |
| Number of present teeth | 1-tooth increments | 1.00 | 0.98 | 1.01 |
| **Daily drinking habits** | **0:No, 1:Yes** | **0.66** | **0.47** | **0.95** |
| Smoking habit | 0:Never smoked |  |  |  |
|  | 1:Used to smoke | 1.11 | 0.79 | 1.55 |
|  | 2:Smoking | 1.17 | 0.71 | 1.93 |
| **Living situation** | **0:Living with someone, 1:Living alone** | **1.34** | **1.02** | **1.77** |
| Education | 1-year increments | 1.01 | 0.96 | 1.06 |
| JST-IC score | 1-score increments | 0.98 | 0.94 | 1.03 |
| MMSE score | 1-score increments | 1.04 | 0.97 | 1.12 |
| Heart disease | 0:No, 1:Yes | 1.11 | 0.79 | 1.56 |
| Diabetes | 0:No, 1:Yes | 1.14 | 0.71 | 1.82 |
| Stroke | 0:No, 1:Yes | 1.02 | 0.59 | 1.74 |
| Serum albumin | 0.1-g/dL increments | 1.17 | 0.67 | 2.05 |
| Hemoglobin A1c | 0.1-% increments | 0.92 | 0.70 | 1.20 |
| **Cohort studies** | **1:Kusatsu Study** |  |  |  |
|  | 2:Otassha Study by Kim et al. | 0.94 | 0.56 | 1.57 |
|  | **3:Otassha Study by Obuchi et al.** | **1.69** | **1.24** | **2.30** |
| Constant |  | 0.02 |  |  |

**Abbreviations:** JST-IC, Japan Science and Technology Agency Index of Competence; MMSE, Mini-Mental State Examination.

Multivariable logistic regression analysis with the presence of poor oral hygiene as the dependent variable. Independent variables that were significantly associated with poor oral hygiene are shown in **bold**.

Supporting Information Table S3: Association between oral dryness and sarcopenia

|  |  |  | 95% confidence intervals | |
| --- | --- | --- | --- | --- |
| Independent variables |  | Odds ratio | Lower limit | Upper limit |
| Sarcopenia | 0:Robust, 1:Sarcopenia | 1.10 | 0.80 | 1.52 |
| Age | 1-year increments | 1.00 | 0.98 | 1.03 |
| Sex | 0:Women, 1:Men | 0.83 | 0.61 | 1.13 |
| Number of present teeth | 1-tooth increments | 0.99 | 0.97 | 1.00 |
| Daily drinking habits | 0:No, 1:Yes | 0.89 | 0.64 | 1.23 |
| Smoking habit | 0:Never smoked |  |  |  |
|  | 1:Used to smoke | 0.87 | 0.64 | 1.20 |
|  | 2:Smoking | 0.80 | 0.49 | 1.31 |
| **Living situation** | **0:Living with someone, 1:Living alone** | **0.76** | **0.59** | **0.98** |
| Education | 1-year increments | 1.00 | 0.96 | 1.05 |
| JST-IC score | 1-score increments | 0.98 | 0.94 | 1.02 |
| MMSE score | 1-score increments | 1.03 | 0.97 | 1.09 |
| Heart disease | 0:No, 1:Yes | 1.35 | 0.99 | 1.85 |
| Diabetes | 0:No, 1:Yes | 1.25 | 0.81 | 1.93 |
| Stroke | 0:No, 1:Yes | 1.21 | 0.73 | 2.01 |
| Serum albumin | 0.1-g/dL increments | 0.88 | 0.53 | 1.46 |
| Hemoglobin A1c | 0.1-% increments | 0.88 | 0.69 | 1.12 |
| **Cohort studies** | **1:Kusatsu Study** |  |  |  |
|  | **2:Otassha Study by Kim et al.** | **2.72** | **1.75** | **4.24** |
|  | **3:Otassha Study by Obuchi et al.** | **2.47** | **1.85** | **3.30** |
| Constant |  | 0.65 |  |  |

**Abbreviations:** JST-IC, Japan Science and Technology Agency Index of Competence; MMSE, Mini-Mental State Examination.

Multivariable logistic regression analysis with the presence of oral dryness as the dependent variable. Independent variables that were significantly associated with oral dryness are shown in **bold**.

Supporting Information Table S4: Association between low occlusal force and sarcopenia

|  |  |  | 95% confidence intervals | |
| --- | --- | --- | --- | --- |
| Independent variables |  | Odds ratio | Lower limit | Upper limit |
| **Sarcopenia** | **0:Robust, 1:Sarcopenia** | **1.63** | **1.10** | **2.40** |
| **Age** | **1-year increments** | **0.95** | **0.92** | **0.97** |
| **Sex** | **0:Women, 1:Men** | **0.46** | **0.31** | **0.69** |
| **Number of present teeth** | **1-tooth increments** | **0.85** | **0.83** | **0.87** |
| Daily drinking habits | 0:No, 1:Yes | 1.05 | 0.71 | 1.56 |
| Smoking habit | 0:Never smoked |  |  |  |
|  | 1:Used to smoke | 1.15 | 0.78 | 1.70 |
|  | 2:Smoking | 0.83 | 0.46 | 1.51 |
| Living situation | 0:Living with someone, 1:Living alone | 1.07 | 0.79 | 1.46 |
| Education | 1-year increments | 1.01 | 0.96 | 1.07 |
| **JST-IC score** | **1-score increments** | **0.94** | **0.89** | **0.99** |
| **MMSE score** | **1-score increments** | **0.93** | **0.86** | **1.00** |
| Heart disease | 0:No, 1:Yes | 1.20 | 0.82 | 1.75 |
| Diabetes | 0:No, 1:Yes | 1.13 | 0.66 | 1.93 |
| Stroke | 0:No, 1:Yes | 1.04 | 0.55 | 1.97 |
| **Serum albumin** | **0.1-g/dL increments** | **0.43** | **0.23** | **0.82** |
| Hemoglobin A1c | 0.1-% increments | 0.85 | 0.63 | 1.15 |
| **Cohort studies** | **1:Kusatsu Study** |  |  |  |
|  | **2:Otassha Study by Kim et al.** | **3.47** | **1.96** | **6.15** |
|  | **3:Otassha Study by Obuchi et al.** | **1.55** | **1.10** | **2.19** |
| Constant |  | 759741.91 |  |  |

**Abbreviations:** JST-IC, Japan Science and Technology Agency Index of Competence; MMSE, Mini-Mental State Examination.

Multivariable logistic regression analysis with the presence of low occlusal force as the dependent variable. Independent variables that were significantly associated with low occlusal force are shown in **bold**.

Supporting Information Table S5: Association between low tongue-lip motor function and sarcopenia

|  |  |  | 95% confidence intervals | |
| --- | --- | --- | --- | --- |
| Independent variables |  | Odds ratio | Lower limit | Upper limit |
| Sarcopenia | 0:Robust, 1:Sarcopenia | 1.28 | 0.92 | 1.78 |
| **Age** | **1-year increments** | **1.06** | **1.03** | **1.08** |
| Sex | 0:Women, 1:Men | 1.20 | 0.85 | 1.70 |
| **Number of present teeth** | **1-tooth increments** | **0.98** | **0.96** | **0.99** |
| Daily drinking habits | 0:No, 1:Yes | 1.00 | 0.70 | 1.42 |
| Smoking habit | 0:Never smoked |  |  |  |
|  | 1:Used to smoke | 1.11 | 0.78 | 1.57 |
|  | 2:Smoking | 1.33 | 0.80 | 2.22 |
| Living situation | 0:Living with someone, 1:Living alone | 0.83 | 0.63 | 1.10 |
| Education | 1-year increments | 0.97 | 0.92 | 1.02 |
| JST-IC score | 1-score increments | 0.96 | 0.92 | 1.00 |
| MMSE score | 1-score increments | 0.99 | 0.93 | 1.05 |
| Heart disease | 0:No, 1:Yes | 1.01 | 0.72 | 1.42 |
| Diabetes | 0:No, 1:Yes | 1.25 | 0.79 | 1.99 |
| **Stroke** | **0:No, 1:Yes** | **2.07** | **1.23** | **3.47** |
| Serum albumin | 0.1-g/dL increments | 0.68 | 0.39 | 1.17 |
| Hemoglobin A1c | 0.1-% increments | 0.99 | 0.77 | 1.29 |
| Cohort studies | 1:Kusatsu Study |  |  |  |
|  | 2:Otassha Study by Kim et al. | 1.13 | 0.71 | 1.79 |
|  | 3:Otassha Study by Obuchi et al. | 0.96 | 0.71 | 1.30 |
| Constant |  | 0.13 |  |  |

**Abbreviations:** JST-IC, Japan Science and Technology Agency Index of Competence; MMSE, Mini-Mental State Examination.

Multivariable logistic regression analysis with the presence of low tongue-lip motor function as the dependent variable. Independent variables that were significantly associated with low tongue-lip motor function are shown in **bold**.

Supporting Information Table S6: Association between low tongue pressure and sarcopenia

|  |  |  | 95% confidence intervals | |
| --- | --- | --- | --- | --- |
| Independent variables |  | Odds ratio | Lower limit | Upper limit |
| **Sarcopenia** | **0:Robust, 1:Sarcopenia** | **2.28** | **1.65** | **3.15** |
| **Age** | **1-year increments** | **1.03** | **1.01** | **1.05** |
| Sex | 0:Women, 1:Men | 0.78 | 0.56 | 1.09 |
| Number of present teeth | 1-tooth increments | 1.01 | 0.99 | 1.02 |
| Daily drinking habits | 0:No, 1:Yes | 1.29 | 0.93 | 1.81 |
| Smoking habit | 0:Never smoked |  |  |  |
|  | 1:Used to smoke | 1.06 | 0.76 | 1.48 |
|  | 2:Smoking | 1.03 | 0.63 | 1.70 |
| Living situation | 0:Living with someone, 1:Living alone | 1.05 | 0.80 | 1.37 |
| Education | 1-year increments | 0.98 | 0.94 | 1.03 |
| **JST-IC score** | **1-score increments** | **0.94** | **0.90** | **0.98** |
| MMSE score | 1-score increments | 0.98 | 0.92 | 1.04 |
| Heart disease | 0:No, 1:Yes | 1.15 | 0.82 | 1.59 |
| Diabetes | 0:No, 1:Yes | 0.99 | 0.63 | 1.56 |
| Stroke | 0:No, 1:Yes | 0.86 | 0.50 | 1.48 |
| **Serum albumin** | **0.1-g/dL increments** | **0.48** | **0.28** | **0.82** |
| Hemoglobin A1c | 0.1-% increments | 0.98 | 0.76 | 1.26 |
| **Cohort studies** | **1:Kusatsu Study** |  |  |  |
|  | 2:Otassha Study by Kim et al. | 0.85 | 0.54 | 1.34 |
|  | **3:Otassha Study by Obuchi et al.** | **0.54** | **0.41** | **0.72** |
| Constant |  | 7.76 |  |  |

**Abbreviations:** JST-IC, Japan Science and Technology Agency Index of Competence; MMSE, Mini-Mental State Examination.

Multivariable logistic regression analysis with the presence of low tongue pressure as the dependent variable. Independent variables that were significantly associated with low tongue pressure are shown in **bold**.

Supporting Information Table S7: Association between low masticatory function and sarcopenia

|  |  |  | 95% confidence intervals | |
| --- | --- | --- | --- | --- |
| Independent variables |  | Odds ratio | Lower limit | Upper limit |
| **Sarcopenia** | **0:Robust, 1:Sarcopenia** | **1.94** | **1.27** | **2.97** |
| Age | 1-year increments | 1.02 | 0.99 | 1.06 |
| Sex | 0:Women, 1:Men | 0.74 | 0.45 | 1.20 |
| **Number of present teeth** | **1-tooth increments** | **0.85** | **0.83** | **0.87** |
| Daily drinking habits | 0:No, 1:Yes | 0.76 | 0.45 | 1.28 |
| Smoking habit | 0:Never smoked |  |  |  |
|  | 1:Used to smoke | 0.90 | 0.55 | 1.46 |
|  | 2:Smoking | 1.21 | 0.62 | 2.38 |
| Living situation | 0:Living with someone, 1:Living alone | 1.39 | 0.97 | 2.00 |
| Education | 1-year increments | 1.01 | 0.94 | 1.08 |
| JST-IC score | 1-score increments | 0.96 | 0.90 | 1.02 |
| MMSE score | 1-score increments | 0.97 | 0.89 | 1.05 |
| Heart disease | 0:No, 1:Yes | 1.16 | 0.74 | 1.83 |
| Diabetes | 0:No, 1:Yes | 0.96 | 0.51 | 1.81 |
| Stroke | 0:No, 1:Yes | 0.81 | 0.39 | 1.69 |
| Serum albumin | 0.1-g/dL increments | 0.91 | 0.44 | 1.91 |
| Hemoglobin A1c | 0.1-% increments | 1.14 | 0.81 | 1.60 |
| Cohort studies | 1:Kusatsu Study |  |  |  |
|  | 2:Otassha Study by Kim et al. | 1.16 | 0.64 | 2.12 |
|  | 3:Otassha Study by Obuchi et al. | 0.87 | 0.58 | 1.32 |
| Constant |  | 1.36 |  |  |

**Abbreviations:** JST-IC, Japan Science and Technology Agency Index of Competence; MMSE, Mini-Mental State Examination.

Multivariable logistic regression analysis with the presence of low masticatory function as the dependent variable. Independent variables that were significantly associated with low masticatory function are shown in **bold**.

Supporting Information Table S8: Association between low of swallowing function and sarcopenia

|  |  |  | 95% confidence intervals | |
| --- | --- | --- | --- | --- |
| Independent variables |  | Odds ratio | Lower limit | Upper limit |
| **Sarcopenia** | **0:Robust, 1:Sarcopenia** | **1.64** | **1.17** | **2.28** |
| Age | 1-year increments | 1.00 | 0.98 | 1.02 |
| Sex | 0:Women, 1:Men | 0.82 | 0.59 | 1.15 |
| Number of present teeth | 1-tooth increments | 0.99 | 0.97 | 1.00 |
| Daily drinking habits | 0:No, 1:Yes | 0.73 | 0.51 | 1.05 |
| Smoking habit | 0:Never smoked |  |  |  |
|  | 1:Used to smoke | 1.21 | 0.86 | 1.69 |
|  | 2:Smoking | 1.10 | 0.66 | 1.84 |
| Living situation | 0:Living with someone, 1:Living alone | 0.84 | 0.64 | 1.11 |
| **Education** | **1-year increments** | **1.07** | **1.01** | **1.12** |
| **JST-IC score** | **1-score increments** | **0.95** | **0.91** | **0.99** |
| MMSE score | 1-score increments | 0.99 | 0.93 | 1.06 |
| Heart disease | 0:No, 1:Yes | 1.10 | 0.79 | 1.54 |
| Diabetes | 0:No, 1:Yes | 1.31 | 0.82 | 2.11 |
| **Stroke** | **0:No, 1:Yes** | **2.25** | **1.36** | **3.72** |
| Serum albumin | 0.1-g/dL increments | 0.67 | 0.39 | 1.16 |
| **Hemoglobin A1c** | **0.1-% increments** | **0.67** | **0.51** | **0.89** |
| Cohort studies | 1:Kusatsu Study |  |  |  |
|  | 2:Otassha Study by Kim et al. | 0.95 | 0.59 | 1.53 |
|  | 3:Otassha Study by Obuchi et al. | 1.21 | 0.89 | 1.63 |
| Constant |  | 21.88 |  |  |

**Abbreviations:** JST-IC, Japan Science and Technology Agency Index of Competence; MMSE, Mini-Mental State Examination.

Multivariable logistic regression analysis with the presence of low of swallowing function as the dependent variable. Independent variables that were significantly associated with low of swallowing function are shown in **bold**.

Supporting Information Table S9: Association between oral hypofunction and sarcopenia

|  |  |  | 95% confidence intervals | |
| --- | --- | --- | --- | --- |
| Independent variables |  | Odds ratio | Lower limit | Upper limit |
| **Sarcopenia** | **0:Robust, 1:Sarcopenia** | **2.17** | **1.52** | **3.09** |
| Age | 1-year increments | 1.01 | 0.98 | 1.03 |
| Sex | 0:Women, 1:Men | 0.92 | 0.65 | 1.30 |
| **Number of present teeth** | **1-tooth increments** | **0.91** | **0.90** | **0.93** |
| Daily drinking habits | 0:No, 1:Yes | 0.76 | 0.53 | 1.09 |
| Smoking habit | 0:Never smoked |  |  |  |
|  | 1:Used to smoke | 1.01 | 0.71 | 1.43 |
|  | 2:Smoking | 0.79 | 0.46 | 1.34 |
| Living situation | 0:Living with someone, 1:Living alone | 0.92 | 0.69 | 1.22 |
| Education | 1-year increments | 1.02 | 0.97 | 1.08 |
| **JST-IC score** | **1-score increments** | **0.92** | **0.87** | **0.96** |
| MMSE score | 1-score increments | 0.99 | 0.92 | 1.06 |
| Heart disease | 0:No, 1:Yes | 1.34 | 0.95 | 1.88 |
| Diabetes | 0:No, 1:Yes | 1.32 | 0.82 | 2.13 |
| **Stroke** | **0:No, 1:Yes** | **1.86** | **1.07** | **3.23** |
| Serum albumin | 0.1-g/dL increments | 0.58 | 0.33 | 1.02 |
| Hemoglobin A1c | 0.1-% increments | 0.89 | 0.67 | 1.16 |
| **Cohort studies** | **1:Kusatsu Study** |  |  |  |
|  | **2:Otassha Study by Kim et al.** | **2.17** | **1.31** | **3.60** |
|  | **3:Otassha Study by Obuchi et al.** | **1.45** | **1.06** | **1.99** |
| Constant |  | 58.09 |  |  |

**Abbreviations:** JST-IC, Japan Science and Technology Agency Index of Competence; MMSE, Mini-Mental State Examination.

Multivariable logistic regression analysis with the presence of oral hypofunction as the dependent variable. Independent variables that were significantly associated with oral hypofunction are shown in **bold**.

Supporting Information References

S10. Sokoloff AJ, Douglas M, Rahnert JA, Burkholder T, Easley KA, Luo Q. Absence of morphological and molecular correlates of sarcopenia in the macaque tongue muscle styloglossus. Exp Gerontol. 2016;84:40–8.

S11. Houston DK, Nicklas BJ, Ding J, Harris TB, Tylavsky FA, Newman AB, et al. Dietary protein intake is associated with lean mass change in older, community-dwelling adults: The Health, Aging, and Body Composition (Health ABC) Study. Am J Clin Nutr. 2008;87:150–5.

S12. Beasley JM, LaCroix AZ, Neuhouser ML, Huang Y, Tinker L, Woods N, et al. Protein intake and incident frailty in the Women's Health Initiative observational study. J Am Geriatr Soc. 2010;58:1063–71.

S13. McLean RR, Mangano KM, Hannan MT, Kiel DP, Sahni S. Dietary protein intake is protective against loss of grip strength among older adults in the Framingham offspring cohort. J Gerontol A Biol Sci Med Sci. 2016;71:356–61.

S14. Rémond D, Machebeuf M, Yven C, Buffière C, Mioche L, Mosoni L, et al. Postprandial whole-body protein metabolism after a meat meal is influenced by chewing efficiency in elderly subjects. Am J Clin Nutr. 2007;85:1286–92.

S15. Suzuki H, Kanazawa M, Komagamine Y, Iwaki M, Amagai N, Minakuchi S. Changes in the nutritional statuses of edentulous elderly patients after new denture fabrication with and without providing simple dietary advice. J Prosthodont Res. 2019;63:288–92.

S16. Suzuki H, Kanazawa M, Komagamine Y, Iwaki M, Amagai N, Minakuchi S. Influence of simplified dietary advice combined with new complete denture fabrication on masticatory function of complete denture wearers. J Oral Rehabil. 2019;46:1100–6.
